# From COVID-19 to Cancer mRNA Vaccines: Moving From Bench to Clinic in the Vaccine Landscape

**DOI:** 10.3389/fimmu.2021.679344

**Published:** 2021-07-07

**Authors:** Chiranjib Chakraborty, Ashish Ranjan Sharma, Manojit Bhattacharya, Sang-Soo Lee

**Affiliations:** ^1^ Department of Biotechnology, School of Life Science and Biotechnology, Adamas University, Kolkata, India; ^2^ Institute for Skeletal Aging & Orthopedic Surgery, Hallym University-Chuncheon Sacred Heart Hospital, Chuncheon, Gangwon-do, South Korea; ^3^ Department of Zoology, Fakir Mohan University, Odisha, India

**Keywords:** mRNA vaccines, mRNA-1273, BNT162b2, mRNA vaccine developers, COVID-19

## Abstract

Recently, mRNA vaccines have become a significant type of therapeutic and have created new fields in the biopharmaceutical industry. mRNA vaccines are promising next-generation vaccines that have introduced a new age in vaccinology. The recent approval of two COVID-19 mRNA vaccines (mRNA-1273 and BNT162b2) has accelerated mRNA vaccine technology and boosted the pharmaceutical and biotechnology industry. These mRNA vaccines will help to tackle COVID-19 pandemic through immunization, offering considerable hope for future mRNA vaccines. Human trials with data both from mRNA cancer vaccines and mRNA infectious disease vaccines have provided encouraging results, inspiring the pharmaceutical and biotechnology industries to focus on this area of research. In this article, we discuss current mRNA vaccines broadly in two parts. In the first part, mRNA vaccines in general and COVID-19 mRNA vaccines are discussed. We presented the mRNA vaccine structure in general, the different delivery systems, the immune response, and the recent clinical trials for mRNA vaccines (both for cancer mRNA vaccines and different infectious diseases mRNA vaccines). In the second part, different COVID-19 mRNA vaccines are explained. Finally, we illustrated a snapshot of the different leading mRNA vaccine developers, challenges, and future prospects of mRNA vaccines.

## Introduction

SARS-CoV-2 has rapidly created a worldwide pandemic, leading to significant health challenges and economic burdens for every country while also causing severe morbidity and mortality. To date, no approved proper treatments or therapeutic choice is available for this virus. Thousands of clinical studies have been registered to discover effective treatments. Simultaneously, this situation has created an urgent need for vaccine development. Vaccines are the most promising solution to fight against the pandemic. Several vaccine candidates are being developed to reduce morbidity and mortality and stop the pandemic. In total, 321 vaccine candidates have been documented from the global R&D setting for the development of COVID-19 vaccines (1). In the developmental phase of the COVID-19 vaccine, a broad range of vaccine approaches is being used, including traditional approaches and next-generation approaches. Several traditional approaches have been used to develop COVID-19 vaccines, such as live coronavirus vaccines, inactivated virus vaccines, and subunit vaccines (2). Similarly, next-generation vaccines for COVID-19 can be divided into protein- or peptide-based vaccines and nucleic acid-based vaccines (3). Nucleic acid-based vaccines are categorized into DNA vaccines and RNA vaccines. Previous studies have shown that conventional vaccine strategies, such as live attenuated vaccines, inactivated vaccines or subunit vaccines, may protect against a range of infectious diseases in the long term (4). In the case of the COVID-19 vaccine, vaccine candidates must be developed more rapidly and in large quantities. Additionally, vaccine candidates must be more effective in the fight against the pandemic. Therefore, mRNA-based vaccines are a more promising choice compared to conventional vaccine strategies because mRNA vaccine candidates have the capacity for rapid development with high effectiveness. These vaccine candidates also have the potential for low-cost manufacturing and safer administration. Therefore, mRNA vaccines have revolutionized the vaccinology field by addressing all of the current challenges (5, 6).

**Figure 1:**
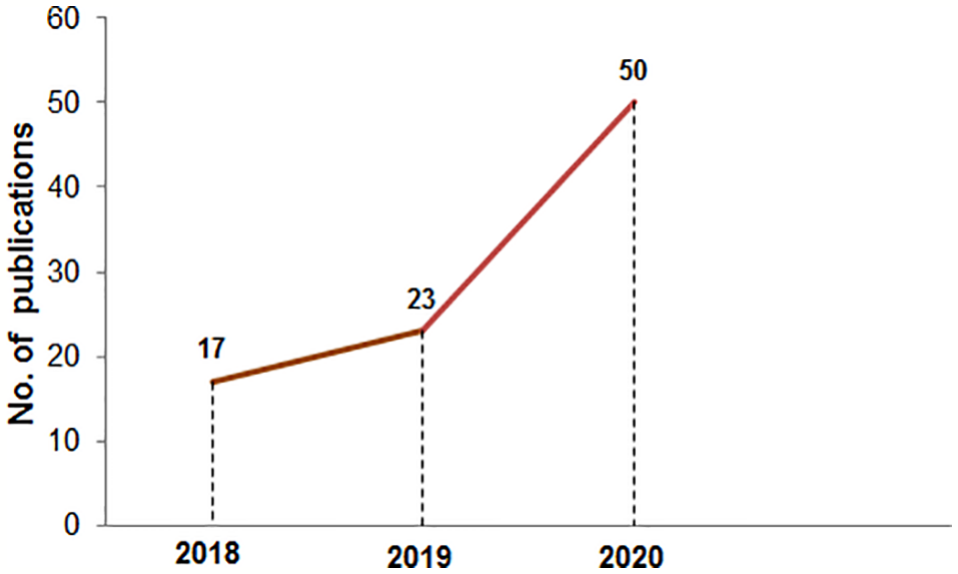
The number of publications in PubMed in the mRNA vaccine area from 2018 to 2020. The PubMed search was performed using the “mRNA vaccine” keyword on 10^th^ Jan 2021.

**Figure 2:**
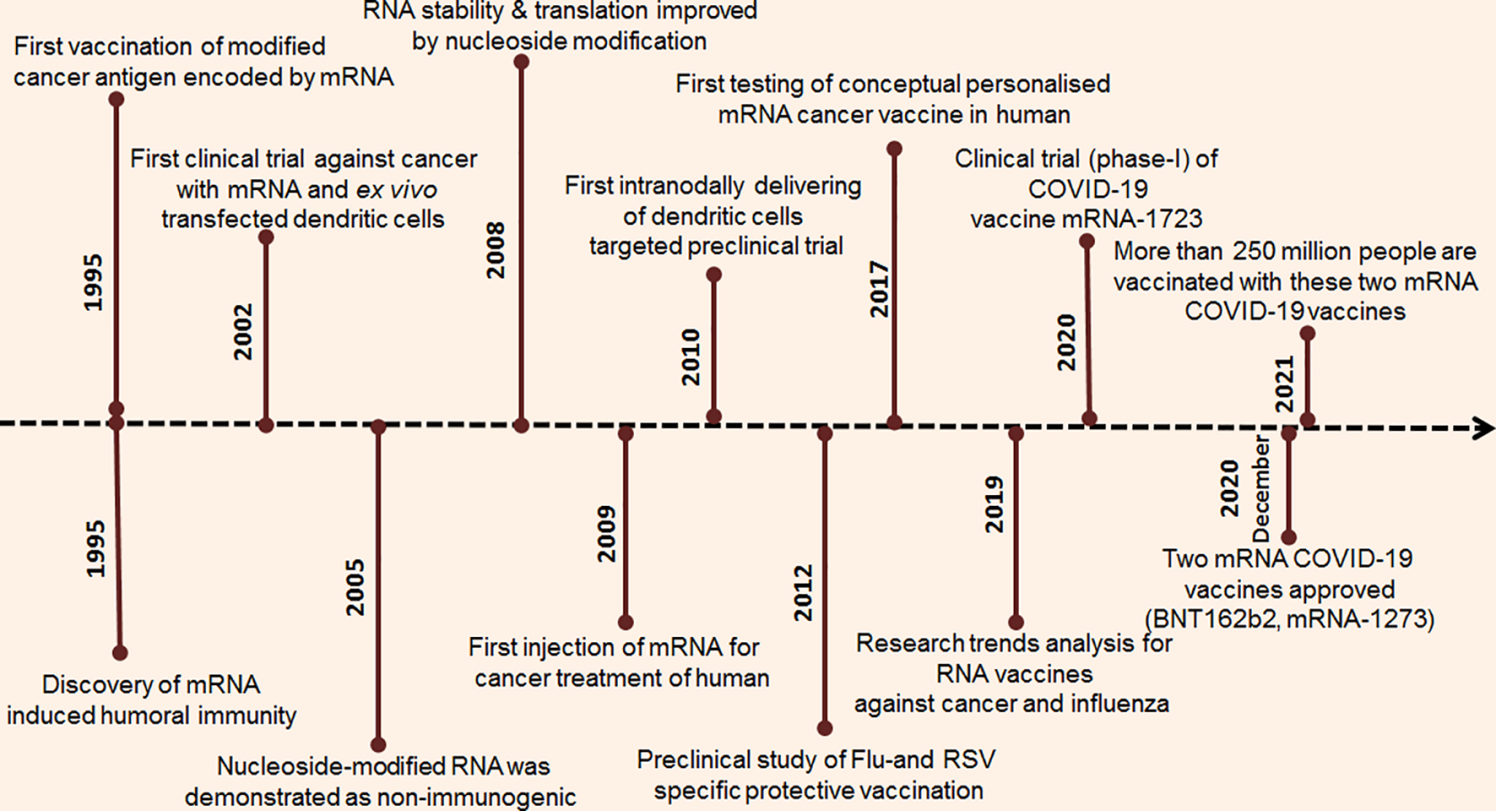
Timeline of the research breakthrough and progression of mRNA vaccine.

The mRNA vaccine development approach is developing quickly (**Figure 1**). Significant research investment in this field has allowed mRNA to become a potential candidate in the immunization landscape. Several major technological innovations have been developed in this area, and pre-clinical research data have been developed and accumulated during the last several years (5, 7). The first successful experiment was published in 1990. In this research, Wolff et al. successfully injected mRNA reporter genes into mouse skeletal muscle cells, and protein production was observed, documenting the first attempt at mRNA *in vivo* expression. This experiment demonstrated a successful method for mRNA vaccine development (8). Subsequently, several studies were performed on mRNA-based therapeutic development (**Figure 2**). Vasopressin mRNA was injected into a rat model to understand the uptake, transport, and expression of this mRNA (9). Several other significant innovations were performed that addressed problems in mRNA vaccine development. One of the important milestones was the assimilation of pseudouridine into mRNA, which provides biological stability and increased translational capacity (10). Another important discovery was optimizing the mRNA coding sequences. In this work, Thess et al. performed sequence engineering of erythropoietin (EPO) mRNA (11). However, codon optimization is not required for mammalian viruses and tumour antigens.

Simultaneously, high-performance liquid chromatography (HPLC) purification of mRNA was performed to identify the contaminants, leading to the generation of pure and therapeutic grade mRNA (12). The first mRNA vaccine entered into a Phase I trial with 13 study subjects in 2011, using a prostate-specific antigen RNA-based vaccine. In this study, mRNA-based prostate-specific antigens were transferred into dendritic cells, which were able to induce *in vitro* T cell-mediated antitumour immune responses (10, 11). Subsequently, several mRNA vaccines have been registered in clinical trials in recent years. Along with the other mRNA vaccine, two COVID-19 mRNA vaccines were developed very rapidly to fight against the pandemic.

In this review, we discuss all forms of mRNA vaccines from COVID-19 to cancer. First, we discuss the preparation of mRNA vaccines in general. Second, we discuss the different delivery systems for mRNA vaccines. Third, we discuss the immune response landscape of mRNA vaccines in general. Fourth, we discuss recent clinical trials of mRNA vaccines. Fifth, we discuss different COVID-19 mRNA vaccines. Finally, we illustrate the different leading mRNA vaccine developers as well as the future prospects of mRNA vaccines.

## *In Vitro* Synthesis of mRNA and Engineering Sequences for mRNA Vaccine Development

**Figure 3:**
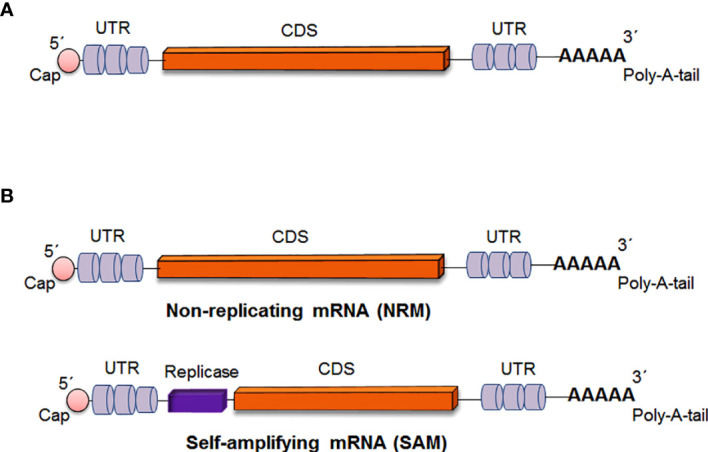
Critical parts of mRNA vaccine construct **(A)** Critical parts of mRNA vaccine constructs in general **(B)** Critical parts of two specific types of mRNA vaccine constructs [Non-replicating mRNA (NRM) vaccine and Self-amplifying mRNA (SAM) vaccine].

In general, mRNA vaccines code the antigen of interest, which contains 5′ and 3′ untranslated regions (UTRs). However, two types of mRNA vaccine constructs are available: nonreplicating mRNA (NRM) vaccine constructs and self-amplifying mRNA (SAM) vaccine constructs. In both cases, there was a universal 5′ cap, 5′ untranslated regions (UTRs), an open reading frame (ORF), 3′ untranslated regions (UTRs) and a 3′ poly(A) tail (**Figure 3**) (5, 7, 13). The ideal structure is described below.

### Ideal Structure of mRNA Vaccine Constructs

#### 5´Cap Structure

CAP structure is an indispensable part of the eukaryotic mRNA. All eukaryotic mRNA has a cap structure, containing an evolutionarily conserved N7-methylated guanosine associated with the first nucleotide of the RNA (14). In the mRNA, the m7GpppN structure is added at the 5´ end as an mRNA cap with numerous functions (15). The cap protects the mRNA from quick degradation. Also, it helps the binding of the initiation factor eIF. There are three types of CAP structures: Cap 0, Cap 1, and Cap2 (16). Cap 0 [m7G(5’)pppN1pN2p] can recruit eIF and helps to prevent mRNA degradation. Cap 0 also helps to stimulate interferon (IFN) mediated responses (17). Cap 1 [m7G(5’)pppN1mpNp] is usually created by the methylation of the 2′-hydroxyl group of the Cap 0. Cap 1 is commonly found in cytoplasmic viruses. Cap 2 [m7G(5’)pppN1mpN2mp] can be generated with an additional 2′-O-methylation of the Cap 1 (6, 14). However, the cap 2 function is still unclear though it has been known that approximately half of all mRNAs possess cap 2. Another cap has also been observed, i.e., m6Am cap. It is reported that cap 0 intermediate and other cap structures are available in eukaryotic mRNAs (18, 19). The cap structure is located on the 5´ end and impacts even protein production (6). In eukaryotes, there are two types of caps: Cap 0 and Cap 1 structures. Following the natural mRNA sequence, adding a regular cap [7-methylguanosine (m7G) cap] is required with the 5´ end of the mRNA sequence, which is an m7GpppN structure (7, 20). Therefore, an analog of a synthetic cap is added to the mRNA during the mRNA vaccine development.

#### 5´ Untranslated Regions

The structures, length, and regulatory elements are significant for mRNA, and these factors regulate the translational efficiency of mRNA.

#### Coding Sequence

The coding sequence of the mRNA vaccine is crucial. Therefore, the coding sequences are modified through codon optimization, which improves the expression of the CDS.

Codon usage is a significant factor that influences protein translation. However, codon optimization is performed during mRNA vaccine CDS design, which can replace rare codons with synonymous codons. In this case, abundantly available codons are used, which are frequent cognate tRNAs accessible in the cytosol. However, this method can augment the production of protein from mRNA (21), and the accuracy of this model has been questioned (22). Another method can be used during mRNA vaccine development that can enhance steady-state mRNA through the enrichment of G:C content (guanine and cytosine content) *in vitro* (23).

#### 3´ Untranslated Regions

The 3’ UTR is a significant part of the mRNA structure that also helps regulate the translational efficiency, similar to 5’ UTRs.

#### 3´ Poly(A) Tail

The 3´ poly(A) tail is important for translation as it protects mRNA molecules and it is also a significant part of the mRNA vaccine structure. The poly(A) tail plays a crucial role in the translation of mRNA by regulating the stability of mRNA (24). Therefore, the best possible length of the poly(A) tail must be included in mRNA through various options, such as using poly(A) polymerase or from the encoding DNA template (25).

### Types of mRNA Vaccine Constructs

#### Non-Replicating mRNA Vaccine Constructs

**Figure 4:**
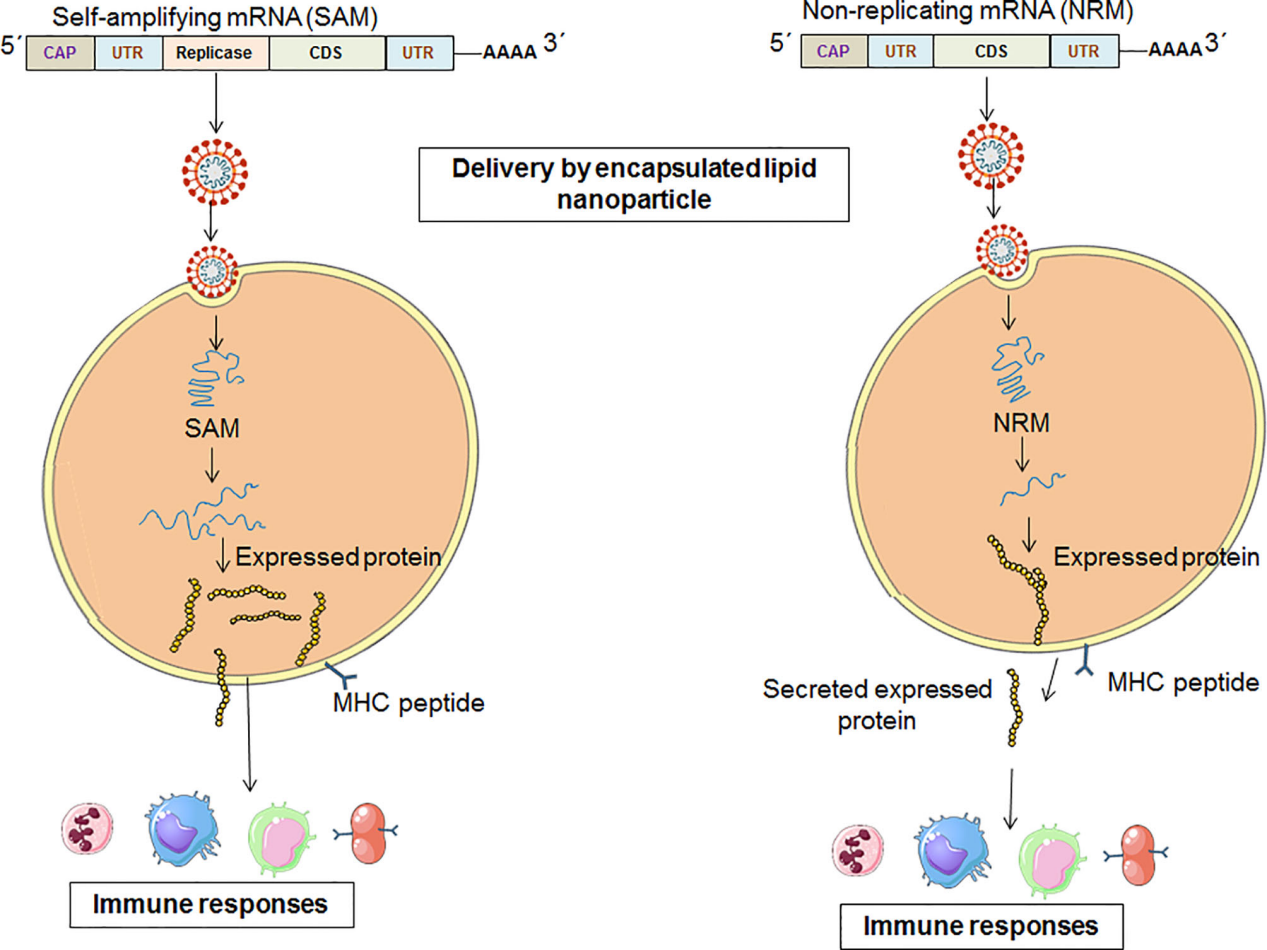
A schematic diagram which explains the pathways from vaccination to immune system activation for the two categories of mRNA vaccine [One for SAM vaccine constructs and other for NRM vaccine constructs].

The NRM is also called the conventional mRNA vaccine (26, 27). The NRM vaccine constructs contain conventional mRNA vaccine sequences, such as the universal 5´ Cap, 5´ untranslated regions (UTRs), an open reading frame (ORF), 3´ untranslated regions (UTRs) and a 3′ poly(A) tail (28, 29). The significant advantage of the NRM vaccine is simplicity. Another advantage of the vaccine is the comparatively small size of the mRNA molecule. Alternatively, limited activity and stability have been observed with the NRM vaccine construct *in vivo*, which is one of the disadvantages of the vaccine (26). However, the optimization of the structural elements of RNA molecules can augment antigen expression and the durability of antigen expression (**Figure 4**) (30).

#### Self-Amplifying mRNA Vaccine Constructs

SAM vaccines are normally constructed and obtained from the engineered RNA of positive-sense single-stranded RNA viruses, such as picornaviruses, flaviviruses, and alphaviruses (31, 32). Alternatively, negative-sense ssRNA viruses have been used to develop mRNA vaccines to protect against measles viruses and rhabdoviruses (33). However, the reverse genetics technique is required to construct the mRNA vaccine from negative-sense RNA genomes. In this case, cell culture-based systems are needed.

The SAM construct contains all of the components of the NRM construct. Additionally, there is an extra component in the SAM construct that encodes an extra replicase component. This component can direct intracellular mRNA amplification (6, 29).

The replication component of the SAM vaccine is generated by substituting viral structural genes that are inserted with antigen-specific genes. Therefore, after delivery, the SAM vaccine components are capable of high levels of amplification in the cytoplasm of target cells, causing high levels of antigen expression (**Figure 4**) (17). The RNA-dependent RNA polymerase (RdRP) complex is in the gene of interest for SAM vaccine constructs as a replicase component (34).

#### Trans-Amplifying mRNA Vaccine

Another type of mRNA vaccine that has recently been studied is called trans-amplifying RNA vaccines. In this type, a replicase can amplify the RNAs “in trans”. The replicase component is offered “in trans”, meaning that two genes act together but on dissimilar RNAs. Self-amplifying RNAs or nonreplicating mRNAs are involved (35). Recently, researchers developed a trans-amplifying RNA system (taRNA system). In this case, replicase-encoding RNA is produced first. Then, a trans-replicon of the antigen of interest is added. Using this influenza vaccine at low doses, animals were protected with the virus (36).

## Different Delivery System for mRNA Vaccine

Efficient mRNA delivery is a significant factor in the therapeutic success of mRNA vaccines. A good delivery system helps mRNA vaccines achieve full therapeutic potential. Naked RNA is prone to nuclease degradation and has difficulty crossing cross the cell membrane because it is negatively charged and is a large molecule. However, several mRNA vaccine delivery strategies have used different delivery strategies: naked mRNA delivery, mRNA delivery through viral vectors, mRNA delivery through polymer-based vectors, mRNA delivery through lipid-based vectors, mRNA delivery through lipid-polymer hybrid nanoparticles, and mRNA delivery through peptide-based vectors (**Table 1**) (16).

**Table 1:** Different delivery system for the different type of mRNA vaccine.

Sl. no	miRNA vaccine type		Route of entry	Disease	Reference
1.	Vector based mRNA	Lipid nanoparticles	Intravenous	Anaemia	(37)
2.				Melanoma	(38)
3.			Subcutaneous	AIDS	(39)
4.			Nasal pumping	Cystic fibrosis	(40)
5.			Intramuscular	Respiratory syncytial virus infection	(41)
6.				ZIKV	(42)
7.				H10N8 and H7N9	(43, 44)
8.		Polymer-based	Subretinal injections	Retina diseases	(45)
9.			Intravenous	Pulmonary vascular disease	(46)
10.				Anaemia, myelodysplasia	(47)
11.			Subcutaneous	AIDS	(48)
12.				Muscle atrophy	(49)
13.		Lipid and polymer hybrid	Intravenous	Ornithine transcarbamylase deficiency	(50)
14.				Lymphoma	(51)
15.		Protamine-formulated	Intradermal	Melanoma	(52)
16.				Prostate cancer	(53)
17.				Non-small cell lung cancer	(54, 55)
18.			Intradermal, intramuscular	Rabies	(56)
19.				Infectious diseases, cancer	(57, 58)
20.			Intradermal	Ovarian cancer	(59)
21.		*Ex vivo* loading of dendritic cells	Subcutaneous	Different tumors	(60)
22.			Intradermal	Acute myeloid leukaemia	(61)
23.	Naked mRNA		Subcutaneous tumors, intranodal	Cervical cancer	(62)
24.			Intradermal	Melanoma	(63)
25.			Intranodal	Cancer	(64)
26.			Intradermal (Electroporation)	–	(65)
27.			Gene gun	Melanoma	(66)
28.			Intradermal (Microneedles)	–	(67)

### Naked mRNA Delivery

The simplest strategy for the delivery of naked mRNA is administration through intramuscular (i.m.) injection (16), while other administration routes include intradermal (i.d.) injection (68) or subcutaneous (s.c.) injection (27). Additionally, researchers have administered mRNA through dissolvable microneedles (RNA patches) (69), which may be an important method for RNA vaccine delivery. However, there are some disadvantages to naked mRNA delivery. The plasma half-life is short, and naked mRNA is prone to ribonuclease degradation. Alternatively, negatively charged mRNA should be neutralized. Otherwise, the mRNA molecule will not be able to pass through cell membranes (70).

### mRNA Delivery Through Viral Vectors

For gene/mRNA delivery, genetically modified viruses have been used for some time. For the delivery of viral RNA, there has been a significant amount of interest in genetic engineering (71). An adeno-associated virus is a viral vector-based delivery system that carries different therapeutic nucleic acid molecules (72). The advantage of RNA viruses is that the virus can replicate and be expressed in the cytoplasm locally and easily. Positive strand RNA viruses can be translated into proteins of interest with the host ribosomal machinery. Therefore, several virus vectors have been developed for mRNA delivery of genes other than adeno-associated viruses, such as flavivirus (73) (e.g., Kunjin virus), picornaviruses (74), and alphaviruses (e.g., Semliki Forest virus and Sindbis) (75). However, there are some disadvantages for mRNA delivery through viral vectors, such as some difficulties with host genome integration and the possibility of host rejection, cytotoxicity, and immunogenicity (76). Wadhwa et al. have also described the drawback of genome integration of mRNA-based vaccine and possible host rejection (16). Ura et al. stated that sometimes genome integration could lead to cancer (77).

### mRNA Delivery Through Lipid Vectors

Lipid-based vectors or lipid-like compounds (lipidoids) are regularly used in mRNA delivery. Naturally occurring lipids and synthetic lipid molecules, such as liposomes or lipid nanoparticles (LNPs), have been used to deliver mRNAs. Liposomes are membrane-bound structures that can be produced through the self-assembly process (78). Dhaliwal et al. used liposomes for mRNA delivery, and cationic liposomes are the preferred system (79). Due to rapid elimination, cationic lipid delivery systems are very challenging (80). However, there are some safety issues of cationic lipids due to their quaternary ammonium head group. Safety issues include immunogenicity and toxicity *in vivo* systems (81) and *in vitro* systems (82). These issues can be solved through the replacement of quaternary ammonium head groups using tripeptide-based lipids (DAO3). Researchers observed that this tripeptide-based lipid has no toxicity *in vivo* or *in vitro* (81).

### mRNA Delivery Through Polymer-Based Vectors

The different polymer-based vectors are used from time to time for mRNA delivery. The first polymer used for mRNA delivery was diethylaminoethyl (DEAE) dextran (83, 84). Later, it was observed that lipid-mediated mRNA delivery is more efficient than the DEAE-dextran-mediated mRNA delivery system (85). However, Siewert et al. observed that charge ratio variation can improve mRNA delivery through the mRNA-DEAE-dextran polyplex system (86). Biodegradable polymers are also used for mRNA delivery, such as biopolymeric nanoparticles [e.g., poly(lactic-co-glycolic acid) (PLGA)]. PLGA is a compatible nanostructure (87, 88), and a PLGA-based mRNA delivery system was developed with tolerable toxicity and substantial transfection efficiency (89).

Alternatively, several multifunctional block copolymers have also been used for mRNA delivery, such as poly(ethylene glycol) methacrylate, dimethylaminoethyl methacrylate (DEAEMA), and DEAEMA-co-n-butyl methacrylate (90). However, due to the high molecular weight of some polymers, polymer-based delivery system design is challenging for mRNA delivery system development (70).

### mRNA Delivery Through Lipid-Polymer Hybrid Nanoparticles

Lipid-polymer hybrid nanoparticles (LPNs) are effective molecules for mRNA delivery. Zhao et al. developed lipid-like nanoparticle TT3-LLN (N1,N3,N5-tris(2-aminoethyl) benzene-1,3,5-tricarboxamide (TT)-derived lipid-like nanomaterial), which was used for efficient mRNA delivery (91). In another study, successful mRNA delivery was performed in the lungs using LPNs, which consist of the degradable polymer poly(β-amino esters) (PBAEs). In this study, PBAEs with PEG and mRNA formulations were developed, which increased *in vitro* potency and serum stability (92). Conversely, using a lipidoid polymer hybrid, co-delivery of siRNA and mRNA was performed, augmenting the co-delivery (93).

### mRNA Delivery Through the Peptide-Based Delivery System

There is increased interest in using peptide-based systems for mRNA delivery due to their versatility (94). Recently, using cell-penetrating peptides (PepFect14), Cerrato et al. performed mRNA delivery for mitochondrial disorders. Researchers have designed a peptide-based delivery technology called mitochondrial peptide-based oligonucleotide technologies, and the technology is promising for treating patients with mitochondrial disorders (95). Conversely, peptide-based delivery systems have some disadvantages, such as targeted cell delivery (59).

## Dendritic Cells: A Potential Target for Delivery of mRNA Vaccines

Dendritic cells are significant antigen-presenting cells (APCs) in the immune system. Dendritic cells instigate the adaptive immune response through antigen processing. Dendritic cells process antigens offered by major histocompatibility complex (MHC) molecules (MHC class I or MHC class II molecules) to T cells (CD^8+^ T cells or CD^4+^ T cells). MHC class I molecules interact with CD^8+^ T cells, and MHC class II molecules interact with CD^4+^ T cells. Furthermore, dendritic cells may process antigens to B-cells to induce the antibody response (96). Wykes and MacPherson (2000) have described that dendritic cells play a significant role in dendritic cells B-cell interaction, thereby activating B-cell to its function and producing antibodies (97). Harvey et al. illustrated a mechanism by which B-cells interact with dendritic cells during the time of antigen processing. This interaction of B-cells with dendritic cells results in the transfer of B-cell receptors with antigen to the APCs. This antigen transfer may result in immunologic response in a more committed way (98). Heesters et al. also described that follicular dendritic cells might play a significant role in the antigen presentation to B-cells. At the same time, they also illustrated that the membrane-bound antigen presentation has a considerable influence on the B-cell activation and its subsequent stages of B-cell responses (99). Therefore, dendritic cells are a significant target for both *ex vivo* and *in vivo* delivery of mRNA vaccines through the transfection process (100). For cancer vaccination, the *ex vivo* dendritic cell loading is being studied to produce cell-mediated immunity (101).

## mRNA Vaccines and the Immune Response Landscape

**Figure 5:**
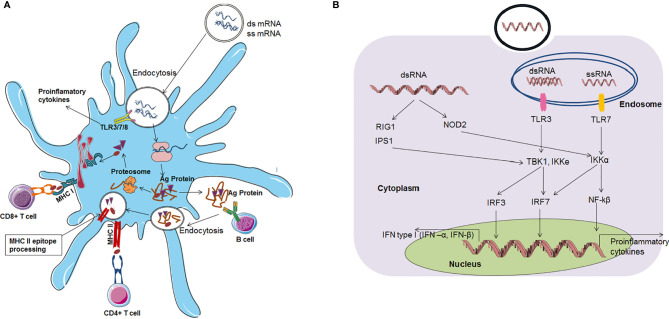
mRNA vaccines based immune response **(A)** mRNA vaccine based adaptive immune response **(B)** mRNA vaccine based innate immune response.

mRNA vaccines can activate both the adaptive immune response and innate immune response (**Figure 5**).

### mRNA Vaccine and the Adaptive Immune Response

The immune response is activated through mRNA vaccines by two routes. First, after vaccination, mRNAs enter the cytoplasm through endocytosis. Dendritic cells are the most important cells for antigen protein presentation. Therefore, these cells are called antigen-presenting cells (APCs) in the immune system. Several mRNAs unite with host cell ribosomes, and translation occurs effectively. After antigen protein synthesis, the antigen is degraded in the cytoplasm into small antigenic peptides *via* the proteasome. Then, these small antigenic peptides are presented *via* the major histocompatibility complex (MHC) to cytotoxic T lymphocytes (CTLs). Alternatively, antigenic proteins can be released by the host cell. Then, these antigenic proteins can be taken up by dendritic cells, and they are degraded and presented to helper T cells and B-cells through MHC. Finally, MHC class I interacts with CD^8+^ T cells, and MHC class II interact with CD^4+^ T cells to activate them. B-cells can also recognize antigen proteins that are released by dendritic cells. Finally, B-cells release antibodies (70, 102).

### mRNA Vaccine and the Innate Immune Response

A self-adjuvant effect has also been noted for mRNA vaccines. In this effect, APCs recognize mRNA, subsequently triggering PRRs (pattern recognition receptors). Pattern recognition receptor members include TLR family members, such as TLR3, TLR7 and TLR8 (58, 103), which are localized in the endolysosomal area of the cell. Receptors in the cytosol can detect nucleic acids in the cytoplasm. ssRNA molecules are recognized by two TLRs: TLR7 and TLR8 receptors. Auridine-rich tetramers are a minimum requirement for both receptors for activation. TLR7-mediated downstream pathway activation aids in type I IFN production (104). AU-rich sequences induce TLR8-mediated downstream pathway activation, leading to a tumour necrosis factor (TNF) response (105). Additionally, dsRNA triggers immune system activation through TLR3 recognition (57, 106). Binding with the TLR3 receptor requires a minimum length of 45 bp dsRNA (107).

Protein families, such as RIG-I, LGP2, and MDA5, function as pattern recognition receptors (108). RIG-I can also recognize dsRNA and ssRNA and activate the downstream pathway, which stimulates IFN production (109–113). mRNA can stimulate the immune response through the TLR pathway, which further stimulates cells to produce augmented amounts of proinflammatory cytokines, type I IFN, and other interferons. These interferons or proinflammatory cytokine molecules degrade RNA, inhibit the translation of mRNA, cause a reduction in CD^8+^ T cells, and ultimately terminate the immune response (5, 7, 10, 114, 115). However, this cascade may produce negative effects for some mRNA vaccines. Therefore, the self-adjuvant property of mRNA vaccines has both disadvantages and advantages.

## Recent Clinical Trial Landscape mRNA Vaccines

Several mRNA vaccines are currently registered for clinical trials. Different mRNA vaccines have been developed against cancer and different types of infectious diseases. mRNA vaccine can balance both adaptive as well as innate immune responses. Therefore, the mRNA vaccine can be used for cancer and infectious diseases (116). These mRNA vaccines have been well studied in animal models and human subjects.

### mRNA Vaccines for Cancer and Their Pre-Clinical and Clinical Update

Different mRNA-based cancer vaccines were designed to target tumor-associated antigens. These antigens are more prevalent in cancerous cells. The majority of cancer vaccines are therapeutic rather than prophylactic (117). These vaccines may stimulate cell-mediated immune responses. Two decades ago, the proposal of RNA-based cancer vaccines was published (118). Recently, several mRNA-based cancer vaccines have been developed that are registered for different phases of clinical trials (**Table 2**). Nevertheless, few trials were terminated due to lack of efficiency, immunogenicity, toxicity, and other side effects. The terminated clinical trials are also listed in **Table 2**. Due to lack of efficiency, one clinical trial (clinical trial no. NCT01582672) was terminated. The trial was conducted using an mRNA vaccine against carcinoma. Similarly, another clinical trial, an mRNA-based prostate cancer vaccine (clinical trial no. NCT01817738) was terminated as the study’s outcome was not as impactful as expected.

**Table 2:** Different mRNA based cancer vaccines which are registered for different phases of the clinical trial.

Vaccine targets	Clinical trials no.	Status (Phase)	Vaccine type	Sponsor/Organization
Prostate cancer	NCT01817738	Terminated (I/II)	RNActivetumour-associated antigen mRNA	CureVac AG
	NCT00831467	Completed (I/II)		
	NCT02140138	Terminated (II)		
	NCT01446731	Completed (II)	Dendritic cell loaded with tumour-associated antigen mRNA	Herlev Hospital
	NCT01197625	Recruiting (I/II)		Oslo University Hospital
	NCT01278914	Completed (I/II)		
	NCT00906243	Terminated (I/II)	RNActivetumour-associated antigen mRNA	University of Florida
Glioblastoma	NCT02649582	Recruiting (I/II)	Dendritic cell electroporated with tumour-associated antigen mRNA	Antwerp University Hospital
	NCT02465268	Recruiting (II)	Dendritic cell loaded with cytomegalovirus antigen mRNA with granulocyte–macrophage colony-stimulating factor protein	University of Florida
	NCT02366728	Active (II)	Dendritic cell loaded with cytomegalovirus antigen mRNA	Duke University
	NCT00626483	Completed (I)		
	NCT02529072	Completed (I)		
	NCT00639639	Active (I)		
	NCT00890032	Completed (I)	Dendritic cell loaded with autologous tumour mRNA	
	NCT02709616	Active (I)	Dendritic cell loaded with tumour-associated antigen mRNA	Guangdong 999 Brain Hospital
	NCT02808364			
	NCT00846456	Completed(I/II)	Dendritic cell loaded with autologous tumour or tumour-associated antigen mRNA	Oslo University Hospital
Pancreatic cancer	NCT00664482	Completed (Not applicable)	Dendritic cell electroporated with autologous tumour mRNA with or without CD40L mRNA	Argos Therapeutics
Melanoma	NCT02035956	Completed (I)	Naked tumour-associated antigen or neo−Ag mRNA	BioNTech RNA Pharmaceuticals GmbH
	NCT01684241			
	NCT02410733	Active (I)	Liposome-complexed tumour-associated antigen mRNA	
	NCT01216436	Terminated	Dendritic cell, matured, loaded with tumour-associated antigen mRNA	Duke University
	NCT01456104	Active (I)	Dendritic cell (Langerhans) electroporated with tumour-associated antigen mRNA	Memorial Sloan Kettering Cancer Center
	NCT01278940	Completed (I/II)	Dendritic cell loaded with autologous tumour or tumour-associated antigen mRNA	Oslo University Hospital
	NCT00961844	Terminated (I/II)		
	NCT02285413	Completed (II)	Dendritic cellelectroporated with tumour or tumour-associated antigen mRNA	Radboud University
	NCT01530698	Completed (I/II)		
	NCT00940004	Completed (I/II)		
	NCT00243529	Completed (I/II)		
	NCT00929019	Terminated (I/II)		
Melanoma	NCT00204516	Completed (I/II)	Autologous tumour mRNA with granulocyte–macrophage colony-stimulating factor protein	University Hospital Tübingen
	NCT00204607	Completed (I/II)	Protamine-complexed tumour-associated antigen mRNA with macrophage colony-stimulating factor protein	
	NCT01983748	Recruiting (III)	Matured Dendritic cell, loaded with autologous tumour RNA	University Hospital Erlangen
	NCT01676779	Completed (II)	Dendritic cellelectroporated with tumour-associated antigen and TriMix mRNA	UniversitairZiekenhuisBrussel
	NCT01066390	Completed (I)		
	NCT01302496	Completed (II)		
Colorectal cancer	NCT00228189	Completed (I/II)	Dendritic cellelectroporated with tumour-associated antigen mRNA	Radboud University
Ovarian cancer	NCT01334047	Terminated (I/II)	Dendritic cell loaded with autologous tumour or tumour-associated antigen mRNA	Oslo University Hospital
	NCT01456065	Unknown	Matured Dendritic cell, loaded with autologous tumour RNA	Life Research Technologies GmbH
Breast cancer z	NCT02316457	Active (I)	Liposome-formulated tumour-associated antigen and neo−antigen mRNA	BioNTech RNA Pharmaceuticals GmbH
	NCT00978913	Completed (I)	Dendritic cellloaded with tumour-associated antigen mRNA	Herlev Hospital
Acute Myeloid Leukemia	NCT00514189	Terminated (I)	Dendritic cell loaded with Acute Myeloid Leukemia lysate and mRNA	MD Anderson Cancer Center
	NCT01734304	Completed (I/II)	Dendritic cell loaded with tumour-associated antigen and cytomegalovirus antigenmRNA	Ludwig-Maximilian-University of Munich
	NCT00510133	Completed (II)	Dendritic cell loaded with tumour-associated antigen mRNA	AsteriasBiotherapeutics
	NCT00965224	Unknown (II)	Dendritic cellelectroporated with tumour-associated antigen mRNA	Antwerp University Hospital
	NCT01686334	Recruiting (II)		
	NCT00834002	Completed (I)		
	NCT03083054	Active (I/II)	Dendritic cell loaded with tumour-associated antigen mRNA	University of Campinas, Brazil
Brain metastases	NCT02808416	Active (I)	Dendritic cell loaded with tumour-associated antigen mRNA	Guangdong 999 Brain Hospital
Non-small-cell lung cancer	NCT01915524	Terminated (I)	RNActivetumour-associated antigen mRNA	CureVac AG
	NCT00923312	Completed (I/II)		
Renal cell carcinoma	NCT00087984	Completed (I/II)	Dendritic cell electroporated with autologous tumour mRNA with or without CD40L mRNA	Argos Therapeutics
	NCT01482949	Terminated (II)		
	NCT01582672	Terminated (III)		
	NCT00678119	Completed (II)		
	NCT00272649	Completed (I/II)		
Mesothelioma	NCT02649829	Recruiting (I/II)	Dendritic cell electroporated with tumour-associated antigen mRNA	Antwerp University Hospital

Slam et al. developed an mRNA vaccine NP (nanoparticle) with a C16-R848 adjuvant for cancer immunotherapy (119). *Ex vivo* dendritic cell loading is currently a method of interest for mRNA-based cancer vaccine development. This vaccine produces cell-mediated immunity efficiently against cancer. Dendritic cell-based mRNA vaccines for cancers have shown promising results in different phases of clinical trials (120). Recently, Ary and colleagues developed mRNA-lipid nanocomplexes that provide strong immune responses to inhibit B16-OVA tumour progression. The mRNA vaccine was tested in tissue culture and mice by direct local injection and increased mouse survival (121). Presently, researchers are trying to develop different mRNA vaccines for different types of cancer.

### mRNA Vaccines for Different Infectious Diseases and Their Pre-Clinical and Clinical Update

Similar to cancer mRNA vaccines, mRNA-based vaccines for different infectious diseases have been studied extensively during the previous two decades. The mRNA-based vaccines developed for different infectious diseases are currently registered for different phases of clinical trials (**Table 3**). In the case of infectious diseases, mRNA vaccines were designed both for therapeutic use and prophylactic use (27). In a Phase-I clinical trial, an mRNA-based vaccine against rabies virus showed that the mRNA could be complexed with protamine. The *in vivo* study showed that it was well-tolerated and safe. Vaccine efficacy depends on the route of administration and the dose. This study demonstrated that the effectiveness of the vaccine was better when administered with a needle-free intramuscular or intradermal device compared to a direct intramuscular or intradermal needle injection (56). Similarly, Bahl and colleagues formulated lipid nanoparticle (LNP)-based mRNA vaccines using the haemagglutinin proteins H10N8 or H7N9, which were tested in ferrets, mice, and nonhuman primates. In a Phase-I clinical trial, the results showed that mRNA vaccines for H10N8 induced robust prophylactic immunity in human volunteers. In this clinical trial, mild or moderate adverse events were noted, while no serious events were documented (43). Presently, several other mRNA vaccines against different infectious diseases are being developed, which are in phases of pre-clinical and clinical trials.

**Table 3:** Different mRNA vaccines for different diseases which are registered for clinical trial.

Vaccine targets	Clinical trials no.	Status (Phase)	Vaccine type	Sponsor/Organization
HIV−1	NCT02888756	Terminated (II)	Dendritic cell; loaded with viral antigenic mRNA with TriMix	Erasmus Medical Center
HIV−1	NCT00833781	Completed (II)	Dendritic cell loaded with viral antigenic mRNA	Massachusetts General Hospital
HIV−1	NCT00381212	Completed (I/II)	Dendritic cellelectroporated with autologous viral antigen and CD40L mRNAs	McGill University Health Centre
Zika Virus	NCT03014089	Completed (I)	Nucleoside-modified viral antigenic mRNA	Moderna Therapeutics
Influenza	NCT03076385	Completed (I)		
HIV−1	NCT02042248	Completed (I)	Dendritic cellelectroporated with autologous viral antigen and CD40L mRNAs	ArgosTherapeutics
	NCT00672191	Completed (II)		
	NCT01069809	Completed (II)		
HIV−1	NCT02413645	Completed (I)	Viral Antigenic mRNA with TriMix	FundacióClínic per la Recerca Biomèdica
Rabies virus	NCT02241135	Completed (I)	Viral antigenic mRNA (RNActive^®^)	CureVac AG

### Toxicity Issues Related to mRNA Vaccines and Some Approved Vaccines scenario

Toxicity is the other side of mRNA vaccines. Sometimes therapeutic nucleosides show toxicity. For example, some nucleoside-based anti-cancer drugs and antivirals drugs containing unnatural nucleoside analogs show toxicity (122–124). During pre-clinical studies, liver toxicity was observed in mRNA therapeutic while delivering them through the lipid nanoparticles. The mRNA therapeutic was developed using a lipid nanoparticle-based delivery system for de Crigler-Najjar syndrome. However, during the delivery of the mRNA vaccine, some delivery system or some formulation might have generated toxicity (125). On the other hand, some systemic adverse events (AEs) were observed for a rabies mRNA vaccine during a human clinical trial. It highlights another side of mRNA, which is the inflammatory nature of the mRNA (56).

In general, toxicity is observed during any vaccine or therapeutics development, especially during pre-clinical studies. It may generally occur due to some issues like developing a delivery system or the formulation of the mRNA vaccine. These are the part of the process of mRNA vaccine development which provides stability/more efficacy to the mRNA molecule. However, if toxicity occurs during development processes, it can be rectified by altering the delivery system or changing the vaccine formulation. Therefore, the therapeutic or vaccine development process is a trial and error method where issues like toxicity may happen during the development process.

The toxicity of an mRNA vaccine is verified through the human clinical trials (Phase-I, Phase-II, and Phase-III), a general rule for the drug development process. If the toxicity is not statistically significant, then only the mRNA vaccine receives approval. Occasionally, in very few cases, toxicity is observed during the clinical trial, which is statistically significant. If the safety profile of an mRNA vaccine is not proper or considerable toxicity of the vaccine is found during the clinical trial, the mRNA vaccine is withdrawn from the clinical trial.

Conversely, there are several success stories of mRNA vaccines. In these cases, three RNA vaccines have shown excellent safety profiles in clinical trials. Recent success stories of two COVID-19 mRNA vaccines (Moderna (mRNA-1273) and Pfizer/BioNTech (BNT162b2)) have demonstrated excellent safety and efficacy profile. Another COVID-19 mRNA vaccine, CureVac, has been registered for Phase-III clinical trial, which CureVac AG has developed. It has shown an ideal safety profile during the Phase-I and Phase-II clinical trials. All the safety profiles of mRNA vaccines are illustrated in **Table 4**. These promising case studies of safety and efficacy profiles will help future manufacturers develop mRNA vaccines with more focus.

**Table 4:** mRNA vaccines (approved/last part of Phase-III trial) which showing very good safety profile.

Sl no.	mRNA vaccine	Disease	Developer	Status	Remark
1.	BNT162b2	COVID-19	BioNTech and Pfizer	The vaccine is approved, several country have started the vaccination program with this vaccine	This vaccine shows excellent safety profile
2.	mRNA-1273	COVID-19	ModernaTX, Inc.	The vaccine is approved, several country have started the vaccination program with this vaccine	This vaccine shows excellent safety profile
3.	CureVac AG	COVID-19	CureVac N.V.	Registered under Phase-III clinical trial (NCT04860258)	The vaccine showed very good safety profile in Phase-I and Phase-II

## COVID-19 mRNA Vaccines

**Figure 6:**
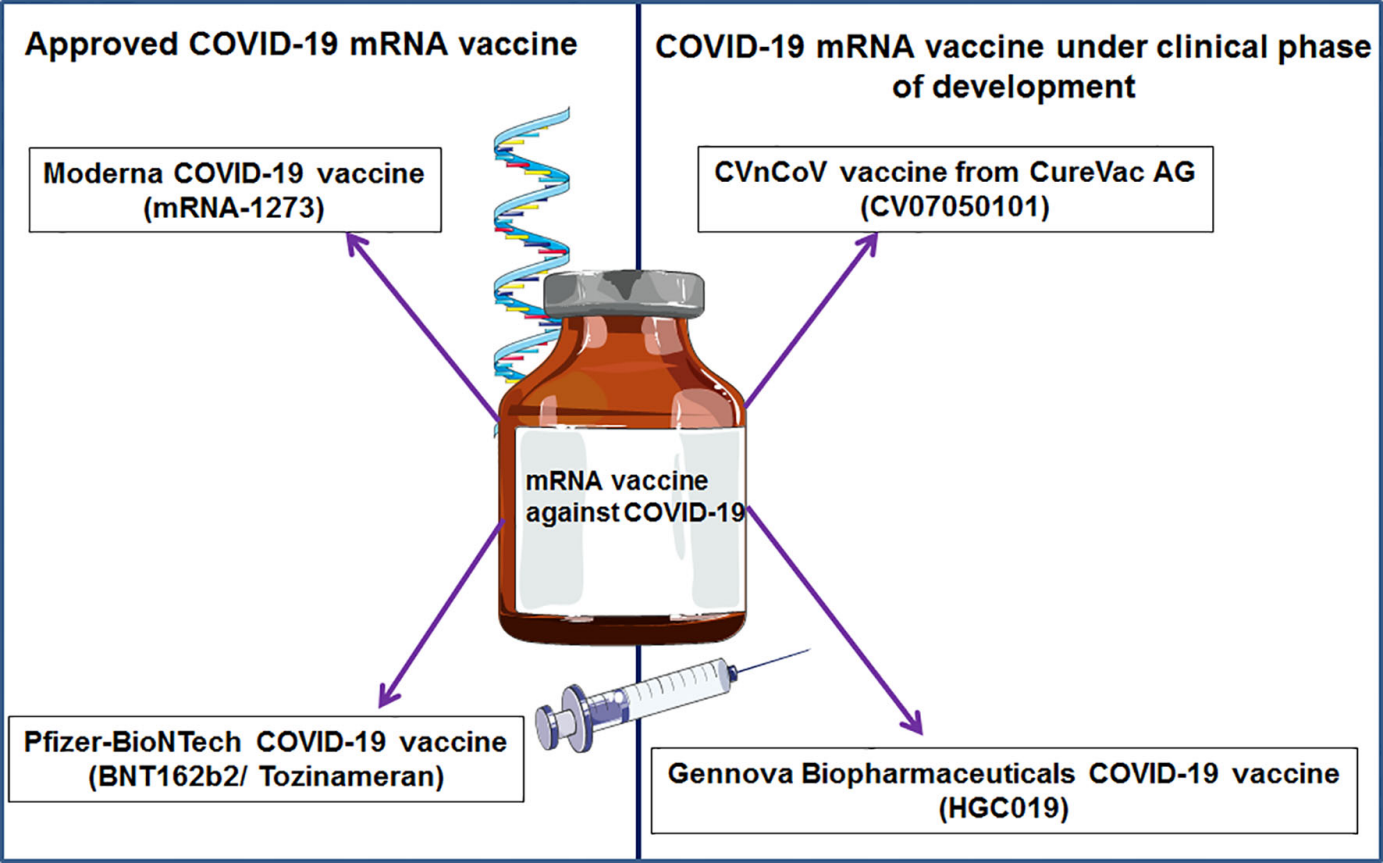
Different significant COVID-19 mRNA vaccines.

Recently, two significant COVID-19 mRNA vaccines have been approved, and others are in development (**Figure 6**). The two approved mRNA vaccine candidates were developed quickly and changed the developmental history of vaccines.

### BNT162b2 mRNA Vaccine From Pfizer and BioNTech

One mRNA-based vaccine was developed jointly by BioNTech and Pfizer against COVID-19, marked as the brand name tozinameran, and the vaccine is also called BNT162b2 (126). It is a modified mRNA vaccine that was developed from the optimized version of the entire spike protein of SARS-CoV-2. This vaccine was formulated with lipid nanoparticles and can be administered through intramuscular injection in two doses, and the two doses can be administered three weeks apart. This vaccine was evaluated through clinical trials conducted in Germany (clinical trial no. NCT04380701) and the USA (clinical trial no. NCT04368728).

In a Phase-I clinical trial, the immunogenicity and safety of the vaccine candidate was evaluated. The results showed neutralizing antibody titres against SARS-CoV-2 and strong antigen-specific T-cell responses (127, 128). The Phase-III clinical trial result was published by Polack et al. (127). In this study, 43,448 human subjects received injections. Among them, 21,728 human subjects received placebo injections, and 21,720 human subjects received BNT162b2 injections. The results show 95% protection for people 16 years of age or older. The vaccine was analysed through a safety profile, which showed headache, fatigue, and mild-to-moderate pain at the injection site (127). This mRNA vaccine was approved by the USA for emergency use. Later, the vaccine was approved by the UK and Canada (126, 129–131).

### mRNA Vaccine (mRNA-1273) From ModernaTX

mRNA-1273 is a mRNA vaccine against COVID-19 that was developed by Moderna and two other research institutes (NIAID and BARDA) of the USA. The vaccine is encapsulated with a lipid nanoparticle carrier that encodes the full-length spike protein of the virus. The vaccine is administered by intramuscular injection. This vaccine is given in two doses that can be administered four weeks apart.

The vaccine was studied through pre-clinical research in nonhuman primate animal models, and the animals received 10 or 100 μg of the vaccine. The results showed that the vaccine augments Th1 (type 1 helper T-cell)-biased CD4 T-cell responses. Additionally, undetectable or very low Th2 or CD8 T-cell responses were noted (132).

A Phase-I clinical trial (NCT04283461) was conducted for this vaccine, which was an open-label and dose-ranging clinical trial performed by the NIH, USA (133).

A Phase-I clinical trial (ClinicalTrials.gov number, NCT04470427) was a randomized, placebo-controlled, observer-blinded trial in which persons 18 years of age or older were vaccinated. The participants received a 1:1 ratio of two intramuscular injections of this vaccine (100 μg). This mRNA vaccine showed 94.1% efficacy (134). This mRNA vaccine was first given emergency use authorization (EUA) in the USA (126, 135) and was later approved by Canada and the UK (135, 136).

### CVnCoV From CureVac AG

CVnCoV is a mRNA vaccine against COVID-19 that was developed by CureVac AG. This vaccine was enrolled in a Phase-IIb/III placebo-controlled, observer-blinded, randomized, multicentre clinical trial (ClinicalTrials.gov number NCT04652102). A total of 36,500 participants were enrolled in this clinical trial. The vaccine was administered through intramuscular (i.m.) injection on day 1 and day 29 (12 µg of vaccine).

### HGC019 From Gennova Biopharmaceuticals

This mRNA vaccine was developed by Gennova Biopharmaceuticals in collaboration with HDT Biotech. This vaccine uses lipid inorganic nanoparticles as a delivery system. This vaccine is currently in a pre-clinical trial stage and will be starting a clinical trial.

## Different Leading mRNA Vaccine Developers

Several leading mRNA vaccine developers have been noted, such as Moderna Therapeutics, Argos Therapeutics, Fundació Clínic per la Recerca Biomèdica, CureVac AG, Gennova Biopharmaceuticals, etc.

### Moderna Therapeutics

This biopharmaceutical company is well known for its mRNA vaccine development. They have developed several vaccines, such as mRNA-1647 against cytomegalovirus, mRNA-1273 against COVID-19, and mRNA-1893 against Zika. mRNA-1647 was registered for a Phase-II clinical trial, and mRNA-1893 was registered for a Phase-I clinical trial. mRNA-1273 received approval for COVID-19.

### Argos Therapeutics

This biopharmaceutical company was founded in 1997 and developed mRNA vaccines against metastatic renal cell carcinoma and HIV-1. Their first vaccine, AGS-003, was developed against metastatic renal cell carcinoma. Their second vaccine, AGS-004, was developed to treat HIV-1 and is registered for a Phase-IIb clinical trial.

### CureVac AG

CureVac AG was founded in 2000, and it is a German biopharmaceutical company that has developed several mRNA vaccines. CV7202 is a mRNA vaccine that uses the rabies virus glycoprotein RABV-G. The vaccine was developed for immunization against the rabies virus. They have also developed CVnCoV against COVID-19, which is registered for Phase-III clinical trials (ClinicalTrials.gov number, NCT04860258). CureVac generated mRNA-based prostate cancer vaccines (CV9103 and CV9104) using RNActive^®^-derived technology (58, 137). CV9103 completed a Phase-I/IIa clinical study for prostate cancer. The vaccine was administered at a dose of 1280 μg (ClinicalTrials.gov number, NCT00906243) (53).

## Current Updates on mRNA Vaccine in Light Of COVID-19 Vaccine and Vaccination

The first approved COVID-19 vaccine is an mRNA vaccine, approved by the USA. Two mRNA vaccines were approved in this direction: Pfizer-BioNTech mRNA-based vaccine (BNT162b2) and Moderna mRNA-based vaccine (mRNA-1273). BioNTech and Pfizer, Inc. developed the first one, and the second one was developed by ModernaTX, Inc. Both of the vaccines were developed at breakneck speed. Presently, both of these vaccines are being used by different countries (Canada, United Kingdom, Israel, and Singapore), including the USA, for mass vaccination to their people (138–140).

## Antigen Activation After mRNA Vaccination: A View in COVID-19 mRNA Vaccination Scenario

Both of the two COVID-19 mRNA vaccines [Moderna (mRNA-1273) and Pfizer/BioNTech (BNT162b2)] have shown excellent safety and efficacy profile and also achieved approximately 90 to 95% efficacy. These vaccines generated considerable neutralizing antibody(nAb) titres during the clinical trial, a study of over 100,000 participants (141). mRNA may act as an immunogen, and adjuvant has immunostimulatory characteristics and can stimulate innate immunity without severe side effects. RNA strands (ssRNA and dsRNA) are recognized by a variety of cytosolic sensors and endosomal sensors. For example, endosomal TLR7 or TLR3 can attach to endosomal ssRNA. While, inflammasome such as PKR, RIG-I, MDA5, and NOD2 may bind to dsRNA/ssRNA present in the cytosol. It can cause further lead to cellular activation. Consequently, various inflammatory mediators and type I IFN are produced (5). Moreover, these mRNA vaccines can activate MDA5 and TLR7, activating the dendritic Cells. Activation of dendritic cells activates the naive T-cells, which helps to generate antibody-secreting plasma cells (5, 141).

The CDC data demonstrated that very few people had side effects after the first doses of BNT162b2 COVID-19 vaccines. It was noted that among 1,893,360 individuals who received first doses of BNT162b2, only 0.2% reported side effects or unpleasant reactions (4393 individuals) (142, 143).

The side effects are recorded as viral enhanced disease (VED) after COVID vaccination with COVID-19 mRNA vaccines. VED is associated with vaccine-associated enhanced respiratory disease (VAERD) and antibody-dependent enhancement (ADE) (142, 143). VAERD is a distinctive medical syndrome, and it is associated with a considerable amount of non-neutralizing antibody production. It usually activates T_H_2 associated immune responses, leading to activation of the complement system. Meanwhile, it also contributes to the generation of the immune complex deposition (144, 145).

On the other hand, during the trial of BNT162b2 (NCT04368728 trial) using 733 patients, malignancies were activated in few patients, which was about 3.9%. In this clinical trial, solid metastatic tumor (4 patients), lymphoma (22 patients), and leukaemia (12 patients) were recorded (128). Hence, several questions are still remains unanswered. For example, does the vaccine is produced after intramuscular or subcutaneous injection leaks to other organs such as the liver or endothelial cells in other sites? Do the antigens spill over to the bloodstream, and which types of cells produce the antigens? These questions need to be resolved immediately to understand more about the COVID mRNA vaccine.

## Challenges and Future Prospects

After the first publications on the delivery of mRNA into animal models, this field has progressed quickly and has shown promise as a next-generation biopharmaceutical for the vaccine development landscape. mRNA vaccines are considered one of the most significant and promising next-generation vaccines due to their rapid development capacity, high potency, safety profile, and low cost of manufacturing. This biopharmaceutical has gained important momentum in the recent few years. Several significant achievements have been observed in the field of mRNA vaccines in the past decade. After the recent launch of two COVID-19 vaccines (mRNA-1273 and BNT162b2), this next-generation mRNA vaccine development landscape has been widely recognized. Human trials with data from both mRNA cancer vaccines and mRNA infectious disease vaccines have provided encouraging results. Therefore, the next few years will be extremely crucial for the development of new mRNA-based therapeutics as the technology is rapidly refined. For the biopharmaceutical industry, investors/sponsor organizations will be more aligned for the generation of mRNA vaccines for new diseases. More public and private partnerships will create more favourable conditions for mRNA-based vaccine development.

However, several points in the area of mRNA vaccine technologies need to be improved. First, there is a need for further advancement of delivery platforms or delivery materials for mRNA vaccines. Second, more extended clinical trials are needed for mRNA vaccines to better understand immunogenicity and safety. Third, the long-term consequences of these vaccine candidates need to be evaluated. Fourth, further optimization is required in the manufacturing processes of mRNA vaccines. Fifth, the stability of formulated mRNA vaccines at normal temperatures is still challenging for vaccine distribution.

The opportunity for mRNA vaccines is considerable, and the future of this vaccine technology is bright. Finally, mRNA vaccines will overcome these obstacles, and more mRNA vaccines will enter the clinic as next-generation vaccines.

## Author Contributions

Conception and design by CC. Writing, reviewing, and/or revising the manuscript by CC, and AS. Review, table, and figure preparations by MB. Overall supervision of the study by CC and S-SL. All authors contributed to the article and approved the submitted version.

## Funding

This study was supported by Hallym University Research Fund.

## Conflict of Interest

The authors declare that the research was conducted in the absence of any commercial or financial relationships that could be construed as a potential conflict of interest.
